# Association of meaning in life with preventive healthcare use among Chinese adults: are there age and gender differences?

**DOI:** 10.1186/s12889-022-14699-0

**Published:** 2022-12-09

**Authors:** Dexing Zhang, Zijun Xu, Zuyao Yang, Weiju Zhou, Peter Man-hin Cheung, Eric Kam-pui Lee, Baoliang Zhong, Dong Xu, Xue Li, Yaojie Xie, Gao Yang, Shuiyuan Xiao, Samuel Yeung-shan Wong

**Affiliations:** 1 JC School of Public Health and Primary Care, The Chinese University of Hong Kong, Hong Kong, China; 2 CUHK Thomas Jing Mindfulness Centre for Research and Training, The Chinese University of Hong Kong, Hong Kong, China; 3 Wuhan Mental Health Center, Huazhong University of Science & Technology, Wuhan, China; 4 School of Health Management, Southern Medical University, Guangzhou, China; 5 Department of Medicine, Li Ka Shing Faculty of Medicine, The University of Hong Kong, Hong Kong, China; 6 Department of Pharmacology and Pharmacy, Li Ka Shing Faculty of Medicine, The University of Hong Kong, Hong Kong, China; 7 School of Nursing, The Polytechnic University of Hong Kong, Hong Kong, China; 8 Department of Sport, Physical Education and Health, Hong Kong Baptist University, Hong Kong, China; 9 Xiangya School of Public Health, Central South University, Changsha, China

**Keywords:** Meaning in life, Preventive healthcare use, Age differences, Gender differences, China

## Abstract

**Background:**

Meaning in life could be of clinical importance in stimulating healthy and preventive behaviors. The study aimed to investigate the association between meaning in life and preventive healthcare use among Chinese adults, and to assess their age and gender differences in the association.

**Methods:**

A cross-sectional online survey was conducted among 1444 adults aged 18–64 years in February 2020 in China. Logistic regression models were employed to examine the association of meaning in life with preventive health checkups and assess their age and gender differences.

**Results:**

The mean score of meaning in life was 5.801 (Standard Deviation = 1.349) out of 7. Each unit increase on the level of meaning in life was associated with 12.2% higher likelihood of using preventive health checkups (any type) (adjusted odds ratio 1.122, 95% confidence interval 1.015–1.241) after adjustment for sociodemographic factors, comorbidity and other psychological health factors. Meaning in life was significantly associated with the uses of X-ray (1.125, 1.010–1.253), B-ultrasound (1.176, 1.058–1.306), and blood testing (1.152, 1.042–1.274). The associations between meaning in life and these types of preventive healthcare increased with age, but there were no gender differences in these associations.

**Conclusion:**

Higher meaning in life was independently related to more preventive health checkups. Strategies to strengthen health education and interventions to improve experience of meaning in life might be an important component to increase preventive healthcare use in China.

## Key messages

What is already known on this topic – Meaning in life could be of clinical importance in stimulating healthy and preventive behaviors.

What this study adds – Meaning in life was independently associated with more preventive health checkups. The association of meaning in life with preventive health checkups was stronger with age, mainly in the uses of X-ray, B-ultrasound, and blood testing, but there were no gender differences in these associations.

How this study might affect research, practice or policy – Future studies are needed to study the potential causal relationship between meaning in life and preventive health checkups among Chinese adults and identify their possible underlying mechanisms.

## Introduction

Meaning in life is one’s fundamental sense of meaning based on an appraisal of his/her significance, directedness, and belongingness of life [1]. People with a higher level of meaning in life show greater happiness, better physical health and reduced health care utilization [2]. Positive relationship has also been revealed between meaning in life and healthier physiological (e.g. reduced inflammatory markers and cardiovascular risk factors) and neurological (e.g. increased insular cortex volume, less cerebral infarcts) states [3–7]. Overall, a growing body of research has suggested that meaning in life is of clinical importance to psychological, physical, and social health and quality of life (QoL) of people [8, 9].

One’s perceived meaning in life may activate goals, self-regulation, and coping with existential challenges [10]. In theory, people with a high level of meaning in life may be more likely to perform preventive health behaviors because they can be more conscious about their health. However, the association of meaning in life with preventive health behaviors, such as receiving health screening, is understudied. Previous studies showed some inconsistent findings of the association between meaning in life and preventive healthcare use [11]. Most of the studies did not adjust for important confounding factors such as biopsychological factors [11, 12]. Few studies have investigated preventive healthcare use in relation to meaning in life among young and middle-aged adults [12, 13]. Little study has assessed age and gender differences in the association of meaning in life with preventive healthcare use [11, 12].

Current knowledge of the association of meaning in life with preventive healthcare use has been predominantly derived from the West, which may not be applicable to those in China, which has a different healthcare system and cultural context from the West. In the traditional Chinese construct of subjective well-being, it has highlighted the importance of sociability and harmony, as in contrast to the more self-construal and individual-oriented culture in the West [14, 15]. Moreover, some studies have underlined the poor health literacy, and suboptimal health education and promotion of healthy behaviors among Chinese population [16, 17]. In this study we examined a cross-sectional online survey of young and middle aged adults in China, to determine the association of perceived meaning in life with preventive healthcare use and explore their age and gender differences. We hypothesized that perceived meaning in life would be associated with utilization of preventive health services among Chinese adults, and the association might vary by age and gender.

## Methods

### Study design and setting

This was a cross-sectional online survey recruiting Chinese adults through the most popular social media channel (WeChat) in China. Convenient samples were recruited in February 2020 through an invitation poster with an introduction of the study background and purpose as well as a scan code which was linked to the online survey. The poster was disseminated by the study investigators to people in their social networks who were invited to further disseminate to their social networks through WeChat.

### Participants and Procedure

Participants were included if they were ≥ 18 years old and could understand Chinese. Upon survey completion, each participant received a health report with brief health advice on diet, physical activity, and respective health advice based on their results. Hotline counselling was provided for any identified health risk. A prize draw incentive of RMB 1–10 was provided as a token of thanks for their time and participation. Each device (e.g. mobile phone and computer) and WeChat account could only complete the survey once to limit multiple filling from one user. Force entry was set up for the key questions. Validity of responses was ensured by checking the time to finish the survey (≥ 250 s), logic check, and consistency of two repeated questions with answers arranged in reverse order. Electronically informed consent was obtained from all participants.

### Measures

#### Meaning in life

Meaning in life was assessed using one item extracted from the validated reliable Chinese Purpose in Life test (CPIL) [18]: “My personal existence is utterly meaningless and without purpose vs. very purposeful and meaningful”. Participants were asked to select a number from a 7-point Likert scale, with 1 denoting the lowest level and 7 denoting the highest level of perceived meaning of existence. This question was also used in our previous study among Chinese [2].

#### Preventive health checkups in the last year

Preventive health checks required answering “yes” to the question “Excluding the need for disease diagnosis and treatment, have you had any health checkups in the past year?”. Participants were then asked what types of preventive health checkups they used, including X-ray, B-ultrasound, Computed Tomography (CT) scan, blood testing, and genetic testing.

#### Demographics, life satisfaction and biopsychosocial health

Age, gender, educational level, marital status, area of living (rural/urban), occupational status, and income were self-reported by the participants. Life satisfaction was collected through asking “All things considered, would you say you are: 1 = very happy, 2 = happy, 3 = not very happy and 4 = not happy at all,” which was used in the World Values Survey by the World Values Survey Association [19]. Depression and anxiety symptoms were assessed by the two-item Patient Health Questionnaire (PHQ-2) and the two-item Generalized Anxiety Disorder Questionnaire (GAD-2), with scores of ≥ 3 indicating positive and higher scores denoting higher severity [20–23]. The Chinese validated three-item UCLA Loneliness Scale (UCLA-3) was used to measure loneliness (range 3–9), with higher scores represented more serious loneliness [24]. The Chinese validated 15-item Patient Health Questionnaire (PHQ-15) was used to measure somatic symptoms [25]. A higher score indicated more somatic symptoms (range from 0 to 30). Information on the number of chronic conditions was also collected. A question on self-rated overall health was measured with a 5-point scale from 1 (poor) to 5 (excellent).

### Data analysis

We described characteristics of participants using mean (standard deviation, SD) for continuous data and percentage (%) for categorical data. Logistic regression models were employed to examine the association between meaning in life (independent variable) and preventive care use (dependent variables), calculating odds ratio (OR) and 95% confidence interval (CI). A p-value of less than 0.05 was considered statistically significant. We further included the socio-demographics (age, gender, marriage, education, occupational status, income, and area living in the past year) and biopsychosocial health and other variables (comorbidity, self-rated health status, life satisfaction, depression (PHQ-2), anxiety (GAD-2), loneliness (UCLA-3), somatic symptoms (PHQ-15)) into the model for adjustment and showed adjusted odds ratio (aOR). We stratified the data by age and gender for subgroup analysis to test their differences in the association of meaning in life with preventive healthcare use, respectively; participants’ age was assorted according to developmental life stages [26, 27]: Emerging Adults (18–24 years), Young Adults (25–44 years) and Middle-Age (45–64 years). We computed a ratio of two ORs (RORs) and tested the differences in the ORs [28]. We further grouped meaning into (1) low, (2) middle, and (3) high levels to determine whether a dose-response relationship exists in the association of meaning in life with preventive healthcare use. All analyses were performed using Stata V.14.0 (Stata Crop, College Station, Texas, USA).

## Results

### Characteristics of the participants

A total of 1444 completed and valid surveys were analyzed after excluding 12 participants aged 65 + years old. The mean age of the participants was 33.6 years (SD 10.1). The majority of them were female (59%), married (59%), employed (70%), having an education level of college or above (88%), perceiving themselves an average income level (60%), and living in urban areas (88%). The details of participants’ characteristics can be seen in Table 1.

**Table 1:** Basic information of participants (*n* = 1444)

Characteristics	Mean (SD) or Number (%)
Age, yr	33.6 (SD 10.1)
Gender	
Male	590 (40.9%)
Female	854 (59.1%)
Marriage	
Married	849 (58.8%)
Single	542 (37.5%)
Cohabiting / separated / divorced / widowed	44 (3.1%)
Remarried	9 (0.6%)
Education	
Primary school and below	7 (0.5%)
Middle school	62 (4.3%)
High school	112 (7.8%)
College degree	215 (14.9%)
Bachelor degree	655 (45.4%)
Postgraduate or above	393 (27.2%)
Occupational status	
Employed	1015 (70.3%)
Unemployed	100 (6.9%)
Student	305 (21.1%)
Unknown	24 (1.7%)
Income level	
Highest / Quite high	98 (6.8%)
High	160 (11.1%)
Average	869 (60.2%)
Low	243 (16.8%)
Lowest / Quite Low	74 (5.1%)
Area living in the past year	
Rural	171 (11.8%)
Urban	1273 (88.2%)
Comorbidity	
No	1057 (73.2%)
Yes	387 (26.8%)
Self-rated health	
Excellent	135 (9.3%)
Very good	480 (33.2%)
Good	508 (35.2%)
Average	310 (21.5%)
Poor	11 (0.8%)
Life satisfaction	
Very happy	118 (8.2%)
Happy	929 (64.3%)
Not very happy	252 (17.5%)
Not happy at all	145 (10.0%)
Depression (PHQ-2)	1.0 (SD 1.3)
Anxiety (GAD-2)	0.8 (SD 1.2)
Loneliness (UCLA-3)	3.9 (SD 1.3)
Somatic symptoms (PHQ-15)	4.0 (SD 4.1)

### Meaning in life

A total of 12 (0.8%), 26 (1.8%), 56 (3.9%), 144 (10.0%), 277 (19.2%), 320 (22.1%) and 609 (42.2%) participants rated a score from 1 (low) to 7 (high) respectively regarding their meaning in life. The mean score was 5.801 (SD = 1.349).

### Association between perceived meaning in life and preventive health care

Univariate analysis showed that each unit increase in meaning in life was associated with a higher likelihood of obtaining any type of health check (OR 1.108, 95% CI 1.026–1.197). In the types of preventive services, the association was significant for X-ray use (1.101, 1.011–1.198), B-ultrasound use (1.191, 1.098–1.293), and blood test taking (1.180, 1.091–1.276), but not significant for CT scan and genetic testing **(**Table 2**)**. After adjustment for all covariates, these significant associations remained, and the corresponding aORs were 1.122 (1.015–1.241) in health check (any type), 1.125 (1.010–1.253) in X-ray, 1.176 (1.058–1.306) in B-ultrasound, and 1.152 (1.042–1.274) in blood testing.

**Table 2:** The relationship between perceived meaning in life and preventive healthcare use

Items	n (%)	Univariate regression		Multivariate regression^a^	
		OR (95% CI)	*p*-value	aOR (95% CI)	*p*-value
Health check (any type)	851 (58.9)	1.108 (1.026, 1.197)	**0.009**	1.122 (1.015, 1.241)	**0.024**
X-ray	454 (31.4)	1.101 (1.011, 1.198)	**0.028**	1.125 (1.010, 1.253)	**0.032**
B-ultrasound	590 (40.9)	1.191 (1.098, 1.293)	**< 0.001**	1.176 (1.058, 1.306)	**0.003**
Blood testing	728 (50.4)	1.180 (1.091, 1.276)	**< 0.001**	1.152 (1.042, 1.274)	**0.006**
CT scan	276 (19.1)	1.060 (0.959, 1.172)	0.253	1.024 (0.906, 1.156)	0.708
Genetic testing	68 (4.7)	0.927 (0.780, 1.100)	0.385	0.964 (0.787, 1.180)	0.721

^a^ Multivariate regression: adjusted for age, gender, marriage, education, job, income, area living in the past year, comorbidity, self-rated health, life satisfaction, depression (PHQ-2), anxiety (GAD-2), loneliness (UCLA-3), somatic symptoms (PHQ-15).

### Subgroup analyses by age and gender

Stratified data by age showed that the association of meaning in life with any of preventive care use seemed greater with age (Table 3); the aOR was 0.892 (0.706–1.127) in Emerging adults, 1.214 (1.064–1.386) in Young adults, and 1.396 (1.002–1.945) in Middle-age adults. Similar associations were observed in the uses of X-ray, B-ultrasound and blood testing, but not in CT scan and genetic testing **(**Table 3**)**.

**Table 3:** Age differences in the relationship between perceived meaning in life and preventive healthcare use

	Emerging adults 18–24 years			Young adults 25–44 years			Middle-age adults 45–64 years		
Items	n (%)	Multivariate regression^a^		n (%)	Multivariate regression^a^		n (%)	Multivariate regression^a^	
		aOR (95% CI)	*p*-value		aOR (95% CI)	*p*-value		aOR (95% CI)	*p*-value
Health check (any type)	127 (40.8)	0.892 (0.706, 1.127)	0.338	550 (62.0)	1.214 (1.064, 1.386)	**0.004**	174 (70.7)	1.396 (1.002, 1.945)	**0.048**
X-ray	47 (15.1)	0.912 (0.661, 1.258)	0.576	300 (33.8)	1.175 (1.023, 1.350)	**0.022**	107 (43.5)	1.487 (1.062, 2.082)	**0.021**
B-ultrasound	51 (16.4)	0.770 (0.568, 1.043)	0.091	403 (45.4)	1.241 (1.084, 1.420)	**0.002**	136 (55.3)	1.436 (1.037, 1.989)	**0.029**
Blood testing	87 (28.0)	0.996 (0.776, 1.278)	0.975	483 (54.5)	1.148 (1.010, 1.305)	**0.035**	158 (64.2)	1.616 (1.159, 2.253)	**0.005**
CT scan	34 (10.9)	0.862 (0.619, 1.201)	0.381	168 (18.9)	1.083 (0.923, 1.270)	0.328	74 (30.1)	1.010 (0.742, 1.374)	0.952
Genetic testing	14 (4.5)	0.847 (0.509, 1.411)	0.524	41 (4.6)	0.967 (0.743, 1.259)	0.802	13 (5.3)	1.227 (0.645, 2.333)	0.533

^a^ Multivariate regression: adjusted for gender, marriage, education, job, income, area living in the past year, comorbidity, self-rated health, life satisfaction, depression (PHQ-2), anxiety (GAD-2), loneliness (UCLA-3), somatic symptoms (PHQ-15).

Further analysis for the subgroups by gender showed that in men increased meaning in life was more strongly associated with health check (any types) (aOR 1.204, 1.028–1.409) and blood testing (aOR 1.212, 1.033–1.422), while in women it was with B-ultrasound (aOR 1.211, 1.045–1.403), but there were no appreciable gender differences in these associations (*P*-values for all RORs > 0.05) (Table 4).

**Table 4:** Gender differences in the relationship between perceived meaning in life and preventive healthcare use

	Men			Women			Gender differences	
Items	n (%)	Multivariate regression^a^		n (%)	Multivariate regression^a^			
		aOR (95% CI)	*p*-value		aOR (95% CI)	*p*-value	ROR	*p*-value
Health check (any type)	344 (58.3)	1.204 (1.028, 1.409)	**0.021**	507 (59.4)	1.061 (0.923, 1.220)	0.405	1.135	0.239
X-ray	192 (32.5)	1.091 (0.926, 1.285)	0.296	262 (30.7)	1.118 (0.963, 1.298)	0.142	0.976	0.829
B-ultrasound	216 (36.6)	1.115 (0.945, 1.314)	0.197	374 (43.8)	1.211 (1.045, 1.403)	**0.011**	0.921	0.464
Blood testing	284 (48.14)	1.212 (1.033, 1.422)	**0.018**	444 (51.99)	1.115 (0.968, 1.283)	0.131	1.087	0.443
CT scan	133 (22.54)	1.105 (0.920, 1.328)	0.284	143 (16.74)	0.923 (0.778, 1.095)	0.356	1.197	0.160
Genetic testing	27 (4.6)	1.141 (0.776, 1.680)	0.502	41 (4.8)	0.915 (0.681, 1.230)	0.556	1.247	0.374

^a^ Multivariate regression: adjusted for age, marriage, education, job, income, area living in the past year, comorbidity, self-rated health, life satisfaction, depression (PHQ-2), anxiety (GAD-2), loneliness (UCLA-3), somatic symptoms (PHQ-15).

### Additional analysis

Meaning in life was further grouped into low, middle, and high levels to examine the dose-response relationships with preventive healthcare use (Table 5). In univariate analysis, OR for health check (any type) in participants with moderate vs. low meaning in life was 1.308 (0.967–1.769), and 1.429 (1.056–1.933) in high vs. low meaning in life respectively. Similar trends were observed in the uses of X-ray, B-ultrasound, and blood testing. After covariate adjustment, there seemed no significant linear associations in different types of health checkups, except for B-ultrasound and blood testing where the corresponding aORs for high vs. low meaning in life were 1.765 (1.175, 2.652) and 1.484 (1.007–2.185), respectively.

**Table 5:** The relationship between perceived meaning in life and preventive healthcare use (by tertile)

Items	Tertile group^a^	n (%)	Univariate regression		Multivariate regression^b^	
			OR (95% CI)	*p*-value	aOR (95% CI)	*p*-value
Health check (any type)	Low	125 (52.5)	1.00		1.00	
	Moderate	353 (59.1)	1.308 (0.967, 1.769)	0.082	1.167 (0.817, 1.667)	0.396
	High	373 (61.3)	1.429 (1.056, 1.933)	**0.021**	1.388 (0.945, 2.039)	0.095
X-ray	Low	62 (26.1)	1.00		1.00	
	Moderate	186 (31.2)	1.285 (0.917, 1.800)	0.146	1.189 (0.812, 1.741)	0.374
	High	206 (33.8)	1.451 (1.038, 2.028)	**0.029**	1.456 (0.967, 2.191)	0.072
B-ultrasound	Low	71 (29.8)	1.00		1.00	
	Moderate	239 (40.0)	1.570 (1.138, 2.168)	**0.006**	1.264 (0.863, 1.851)	0.230
	High	280 (46.0)	2.002 (1.453, 2.757)	**< 0.001**	1.765 (1.175, 2.652)	**0.006**
Blood testing	Low	95 (39.9)	1.00		1.00	
	Moderate	302 (50.6)	1.541 (1.136, 2.091)	**0.005**	1.234 (0.862, 1.766)	0.251
	High	331 (54.4)	1.792 (1.321, 2.430)	**< 0.001**	1.484 (1.007, 2.185)	**0.046**
CT scan	Low	43 (18.1)	1.00		1.00	
	Moderate	108 (18.1)	1.002 (0.678, 1.480)	0.994	0.883 (0.572, 1.364)	0.575
	High	125 (20.5)	1.171 (0.797, 1.720)	0.420	0.963 (0.606, 1.531)	0.875
Genetic testing	Low	14 (5.9)	1.00		1.00	
	Moderate	26 (4.4)	0.729 (0.374, 1.421)	0.353	0.668 (0.317, 1.409)	0.289
	High	28 (4.6)	0.771 (0.399, 1.492)	0.440	0.866 (0.387, 1.940)	0.727

^a^Meaning in life was classified into three categories and its classification of low, moderate, and high levels were: (1) low: score 1–4, (2) moderate: score 5–6, (3) high: score 7

^b^Multivariate regression: adjusted for age, gender, marriage, education, job, income, area living in the past year, comorbidity, self-rated health, life satisfaction, depression (PHQ-2), anxiety (GAD-2), loneliness (UCLA-3), somatic symptoms (PHQ-15).

## Discussions

In our cross-sectional online survey of Chinese adults, higher levels of perceived meaning in life were associated with a higher likelihood of obtaining preventive healthcare use. It was found that participants with higher levels of meaning in life were more likely to obtain an X-ray test, B-ultrasound, or blood testing. The associations of meaning in life with these types of preventive healthcare services increased with age and there were no gender differences in the associations.

To the best of our knowledge, this is the first study to examine the association of perceived meaning in life with preventive healthcare use among Chinese, and the first study to look into age and gender differences in the associations. The findings are important and have clinical implications in improving uptake of preventive healthcare services. Meaning in life can positively activate one’s behaviors towards one’s short- and long-term goals [29]. People who have meaningful living can be more engaged with daily activities and selecting activities such as use of preventive healthcare that match one’s long-term aims [30]. It is likely that by boosting one’s meaning in life, people are more likely to take other preventive healthcare services such as preventive cancer screening.

Two studies have been found to investigate the association of meaning in life with preventive healthcare use among young and middle aged women (age from 20 to 42 years) for breast cancer screening [11, 13]. It showed a significant relationship between purpose in life and breast health behaviors in Anglo women but not in Hispanic women. However, positive psychological factor associated with more preventive healthcare use is consistent with previous studies that conducted in middle and old age [12, 13]. A study of a representative sample of American older adults aged 50 + showed that each unit increase in purpose in life (on a six-point scale) was associated with a higher likelihood that people would engage in recommended preventive health care services, such as obtaining a cholesterol test (aOR 1.18, 1.08–1.29) or colonoscopy (aOR 1.06, 0.99–1.14). Another study conducted among 162 members aged 75–95 of the Terman Study of the Gifted in the US showed that higher purpose in life was associated with more regular checkups [12]. Life purpose and meaning in life are slightly different, but life purpose is one of key factors contributing to a meaningful life. In our study we included more health checkup types such as B-ultrasound and blood testing, and the significant associations with meaning in life were remained after adjustment for more important covariates, such as psychological factors. Our data from China have contributed to the literature.

The findings from this study may help explain the growing body of research that has linked higher meaning in life with greater wellbeing, both physically [8] and psychologically [31]. For example, a meta-analysis which included 66 papers reported that meaning in life and physical health formed weak-to-moderate associations and the strongest associations were found for subjective indicators of physical health [8]. In the context of our study, people with higher levels of meaning in life may have healthier lifestyles (e.g., having better nutritional and dietary habits [32], engaging in more exercise and relax [33, 34]) and acquire more regular health checkups because they have a greater meaning to live, which gives them more incentive to take preventive measures that may seem costly, time consuming, and against instant hedonism. All of these activities may be prompted by an overarching outlook in which life itself is greatly valued.

Our study found that the association of meaning in life with preventive healthcare use was stronger with age, even though increased age has a significantly negative effect on life satisfaction [35] and mental health outcomes (e.g., depression [36]) during the COVID-19 epidemic in many previous studies. This might be because people at later life stages generally reported a greater presence of meaning in their lives, while those at earlier life stages remained searching for their life meaning [35]. Meaning in life is considered modifiable factor and preventive health behavior allow detection of diseases in early phase. Paying more attention to improve experience of meaning in life among young and middle-aged adults is important to promote preventive healthcare use, and thereby prevention of complications and reduction in healthcare cost in their late-life. This is especially important in countries with limited health resources, where the healthcare system is overburdened due to rapidly aging population and widespread inequality in healthcare access [16, 17].

Gender differences in the association of meaning in life with preventive healthcare use is not well studied [13]. In previous literature men with increased meaning in life were more likely to receive a prostate examination (OR 1.31, 1.18–1.45), and women were more likely to receive a mammogram/X-ray (OR 1.27, 1.16–1.39) or pap smear (1.16, 1.06–1.28), but their gender differences have not been evaluated [13]. Our study showed no significant gender differences in the association of meaning in life with preventive healthcare use, suggesting that improving experience of meaning in life to promote preventive healthcare use is equally important for men and women.

Our study identified a linear association between meaning in life and preventive healthcare use in univariate analysis. The main three preventive healthcare use including X-ray, B-ultrasound, and blood testing showed such a linear association. These linear trends seemed to be non-significant after adjustment for all covariates, except for B-ultrasound and blood testing. The high meaning group was associated with B-ultrasound but the moderate meaning group was not. This may be due to the small number of participants in these groups, where their p values for high vs. low meaning in life were approaching statistical significances. We need further studies to focus on this issue in China, including more participants to clarify linear associations between meaning in life and different types of preventive healthcare use.

The current study did not find a significant association of meaning in life with CT scan or genetic testing. One possible explanation is related to their costs; in the public healthcare system, CT scan and genetic testing are categorized under private services and their costs are approximately 3–10 times higher than the other three preventive healthcare services, depending on the body parts involved and the items covered. Furthermore, CT scan and genetic testing are less common or necessary than B-ultrasound and blood testing in a general preventive healthcare service. In general, CT scan will be reserved for circumstances in which there is diagnostic uncertainty (e.g., cancer), while genetic testing would be performed to learn a current or future pregnancy whether or not will be affected by a genetic illness.

### Strength and limitations

The current study included a large sample of Chinese population and its strict data validation had ensured data quality. Using a convenience sample method, the well-educated participants probably had a good overall understanding of perceived meaning in life and preventive health checkups. The multivariate analyses had included many important covariables for adjustment such as psychological factors including depression and anxiety, and thus the confounding effect would be minimized. At the same time, the study has several limitations. First, the included sample was self-selected and was biased towards individuals with high education level in urban areas because of the use of online collection method during COVID-19. Therefore, the generalizability of our results to other populations (e.g., low education level, rural areas) was not known. However, during lockdown due to COVID-19 outbreak, other data collection methods such as face-to-face interview were considered infeasible. Second, our data were collected in February 2020; we did not know whether or not the COVID-19 outbreak (since December 2019) might have interfered in the use of the preventive healthcare checkups. These are not taken into account in the models. However, it might be likely the interference would not change the founded significant associations as these health checkups were not COVID-19 specific by that time. Third, causal relationship cannot be confirmed due to our cross-sectional design. Fourth, the study had only applied a single item to test meaning in life due to that long questionnaire was not feasible in online survey. However, a previous systematic review with meta-analysis [36] found that results remained the same regardless of which questionnaire or if only single-item measure was applied. We had also used this question among another Chinese population and observed its significant associations with physical health, happiness, and healthcare utilization, suggesting it was acceptable to adopt this one-item question [2]. This supports the suitability of using one item to test one’s perceived meaning in life in short surveys.

### Implications

First, further studies may consider studying the potential causal relationship between meaning in life and preventive healthcare use and identify possible underlying mechanisms, to shed light on exploration of meaning in life as one of the potential interventional targets for improved preventive healthcare service uptake. Second, due to the self-reporting nature of the survey, it was unknown if the used preventive health checkups (e.g., X-ray and CT scan) were evidence-based or not. It should note that use of non-evidence based preventive tests may cause harm and wastage of healthcare resources, instead of being cost-saving. More education and interventions should be taken timely if non-evidence based preventive health checkups were taken among the population with high level of meaning in life. At the same time, health benefits could be further studied after uptake of the needed preventive healthcare services.

## Conclusion

This study demonstrated independent positive association of meaning in life with preventive health checkups, mainly in the uses of X-ray, B-ultrasound, and blood testing. The association of meaning in life with preventive health checkups was stronger with age. There were no significant gender differences in these associations, and thus strategies to improve experience of meaning in life might be of equal importance to increase preventive healthcare service use in men and women in China.

## Data Availability

The data that support the findings of this study are available from the corresponding author, [Samuel YS Wong, email: yeungshanwong@cuhk.edu.hk], upon reasonable request.

## References

[1] Schnell T (2009). The sources of meaning and meaning in Life Questionnaire (SoMe): relations to demographics and well-being. J Posit Psychol.

[2] Zhang D, Chan DC, Niu L, Liu H, Zou D, Chan AT, Gao TT, Zhong B, Sit RW, Wong SY (2018). Meaning and its association with happiness, health and healthcare utilization: a cross-sectional study. J Affect Disord.

[3] Costa T, Suardi AC, Diano M, Cauda F, Duca S, Rusconi ML, Sotgiu I (2019). The neural correlates of hedonic and eudaimonic happiness: an fMRI study. Neurosci Lett.

[4] Lewis GJ, Kanai R, Rees G, Bates TC (2014). Neural correlates of the ‘good life’: eudaimonic well-being is associated with insular cortex volume. Soc Cogn Affect Neurosci.

[5] Urry HL, Nitschke JB, Dolski I, Jackson DC, Dalton KM, Mueller CJ, Rosenkranz MA, Ryff CD, Singer BH, Davidson RJ (2004). Making a life worth living: neural correlates of well-being. Psychol Sci.

[6] Waytz A, Hershfield HE, Tamir DI (2015). Mental simulation and meaning in life. J Pers Soc Psychol.

[7] Yu L, Boyle PA, Wilson RS, Levine SR, Schneider JA, Bennett DA (2015). Purpose in life and cerebral infarcts in community-dwelling older people. Stroke.

[8] Czekierda K, Banik A, Park CL, Luszczynska A (2017). Meaning in life and physical health: systematic review and meta-analysis. Health Psychol Rev.

[9] Chui RC (2018). The role of meaning in life for the quality of life of community-dwelling Chinese elders with low socioeconomic status. Gerontol Geriatr Med.

[10] Vos J, Vitali D (2018). The effects of psychological meaning-centered therapies on quality of life and psychological stress: a metaanalysis. Palliat Support Care.

[11] Wells JN, Bush HA, Marshall D (2002). Purpose-in-life and breast health behavior in hispanic and anglo women. J Holist Nurs.

[12] Holahan CK, Suzuki R (2006). Motivational factors in health promoting behavior in later aging. Act Adapt Aging.

[13] Kim ES, Strecher VJ, Ryff CD (2014). Purpose in life and use of preventive health care services. Proc Natl Acad Sci U S A.

[14] Yang D, Zhou H (2017). The comparison between chinese and western well-being. Open J Social Sci.

[15] Ryff CD, Singer BH (2008). Know thyself and become what you are: a eudaimonic approach to psychological well-being. J Happiness Stud.

[16] Li X, Krumholz HM, Yip W, Cheng KK, De Maeseneer J, Meng Q, Mossialos E, Li C, Lu J, Su M (2020). Quality of primary health care in China: challenges and recommendations. Lancet.

[17] Wang XQ, Chen PJ (2014). Population ageing challenges health care in China. Lancet.

[18] Shek DT (1988). Reliability and factorial structure of the chinese version of the purpose in Life Questionnaire. J Clin Psychol.

[19] Easterlin RA, McVey LA, Switek M, Sawangfa O, Zweig JS (2010). The happiness-income paradox revisited. Proc Natl Acad Sci U S A.

[20] Tong X, An D, McGonigal A, Park SP, Zhou D (2016). Validation of the generalized anxiety Disorder-7 (GAD-7) among chinese people with epilepsy. Epilepsy Res.

[21] Wang W, Bian Q, Zhao Y, Li X, Wang W, Du J, Zhang G, Zhou Q, Zhao M (2014). Reliability and validity of the chinese version of the patient health questionnaire (PHQ-9) in the general population. Gen Hosp Psychiatry.

[22] Löwe B, Kroenke K, Gräfe K (2005). Detecting and monitoring depression with a two-item questionnaire (PHQ-2). J Psychosom Res.

[23] Donker T, van Straten A, Marks I, Cuijpers P (2011). Quick and easy self-rating of generalized anxiety disorder: validity of the dutch web-based GAD-7, GAD-2 and GAD-SI. Psychiatry Res.

[24] Russell DW (1996). UCLA Loneliness Scale (Version 3): reliability, validity, and factor structure. J Pers Assess.

[25] Lee S, Ma YL, Tsang A (2011). Psychometric properties of the chinese 15-item patient health questionnaire in the general population of Hong Kong. J Psychosom Res.

[26] Erikson EH (1994). Identity: youth and crisis.

[27] Arnett JJ (2000). Emerging adulthood. A theory of development from the late teens through the twenties. Am Psychol.

[28] Altman DG, Bland JM (2003). Interaction revisited: the difference between two estimates. BMJ.

[29] McKnight PE, Kashdan TB (2009). Purpose in life as a system that creates and sustains health and well-being: an integrative, testable theory. Rev Gen Psychol..

[30] Scheier MF, Wrosch C, Baum A, Cohen S, Martire LM, Matthews KA, Schulz R, Zdaniuk B (2006). The life engagement test: assessing purpose in life. J Behav Med.

[31] Steger MF (2012). Experiencing meaning in life: optimal functioning at the nexus of well-being, psychopathology, and spirituality. The human quest for meaning: theories, research, and applications.

[32] Piko BF, Brassai L (2009). The role of individual and familial protective factors in adolescents’ diet control. J Health Psychol.

[33] Brassai L, Piko BF, Steger MF (2015). A reason to stay healthy: the role of meaning in life in relation to physical activity and healthy eating among adolescents. J Health Psychol.

[34] Holahan CK, Holahan CJ, Velasquez KE, Jung S, North RJ, Pahl SA (2011). Purposiveness and leisure-time physical activity in women in early midlife. Women Health.

[35] Steger MF, Oishi S, Kashdan TB (2009). Meaning in life across the life span: levels and correlates of meaning in life from emerging adulthood to older adulthood. J Posit Psychol.

[36] Cohen R, Bavishi C, Rozanski A (2016). Purpose in life and its relationship to all-cause mortality and cardiovascular events: a meta-analysis. Psychosom Med.

